# Nobiletin enhances plasma Interleukin‐6 and C‐X‐C motif chemokine ligand 1 levels that are increased by treadmill running

**DOI:** 10.1002/fsn3.2844

**Published:** 2022-03-24

**Authors:** Toshihide Suzuki, Makoto Shimizu, Yoshio Yamauchi, Ryuichiro Sato

**Affiliations:** ^1^ Nutri‐Life Science Laboratory Department of Applied Biological Chemistry Graduate School of Agricultural and Life Sciences The University of Tokyo Tokyo Japan; ^2^ Food Biochemistry Laboratory Department of Applied Biological Chemistry Graduate School of Agricultural and Life Sciences The University of Tokyo Tokyo Japan

**Keywords:** C‐X‐C motif chemokine ligand 1, epinephrine, exercise, Interleukin‐6, nobiletin

## Abstract

Exercise increases the muscular secretion of Interleukin‐6 (IL‐6), which is partially regulated by β2‐adrenergic receptor signaling. Nobiletin is a polymethoxyflavone (PMF) found in citrus fruits that induces the secretion of IL‐6 from C2C12 myotubes, but it remains unclear whether nobiletin promotes IL‐6 secretion during exercise. The aim of this study was to clarify the effects of nobiletin on IL‐6 secretion during exercise. Nobiletin and epinephrine were found to synergistically increase IL‐6 secretion from differentiated C2C12 cells, which was suppressed by the inhibition of adenylyl cyclase (AC) or protein kinase A (PKA). Treadmill running for 60 min increased plasma levels of IL‐6, epinephrine, and norepinephrine in rats. Nobiletin (5 mg/kg) orally administered 30 min before running increased plasma IL‐6 levels further, although it did not increase plasma epinephrine and norepinephrine. In a similar manner to IL‐6, nobiletin and epinephrine synergistically increased the secretion of C‐X‐C motif chemokine ligand 1 (CXCL‐1) from C2C12 cells, or the increase in plasma CXCL‐1 was enhanced by nobiletin after treadmill running of rats. Our results suggest that nobiletin promotes IL‐6 and CXCL‐1 secretion from skeletal muscle by synergistic enhancement of the PKA pathway in β2‐adrenergic receptor signaling.

## Graphical abstract

Nobiletin enhances plasma Interleukin‐6 (IL‐6) and C‐X‐C motif chemokine ligand 1 (CXCL‐1) levels increased by treadmill running. This may be because of synergistic activation of the protein kinase A–cAMP response element binding protein (PKA–CREB) pathway by increased plasma catecholamines after exercise and by nobiletin administration, which promotes IL‐6 and CXCL‐1 secretion from skeletal muscle into blood.

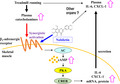

## 1 INTRODUCTION

Myokines are cytokines secreted by skeletal muscle that exert physiological effects not only on skeletal muscle but also on various organs such as the brain, liver, and adipose tissue via the blood (Giudice & Taylor, 2017). Interleukin‐6 (IL‐6) is one of the most investigated myokines, and exercise increases its levels in the plasma (Fischer, 2006). The function of IL‐6 as a myokine is to regulate energy metabolism through the promotion of glucose uptake in skeletal muscle, gluconeogenesis in the liver, and lipolysis in adipose tissue (Pedersen & Febbraio, 2008). It also promotes myoblast proliferation and differentiation (Muñoz‐Cánoves et al., 2013). IL‐6 synthesis and secretion are promoted by physical stimuli such as skeletal muscle contraction and hyperthermia (Furuichi et al., 2018; Welc et al., 2012), and pharmacological stimuli such as epinephrine and lipopolysaccharide (Frost et al., 2004).

**FIGURE 1:**
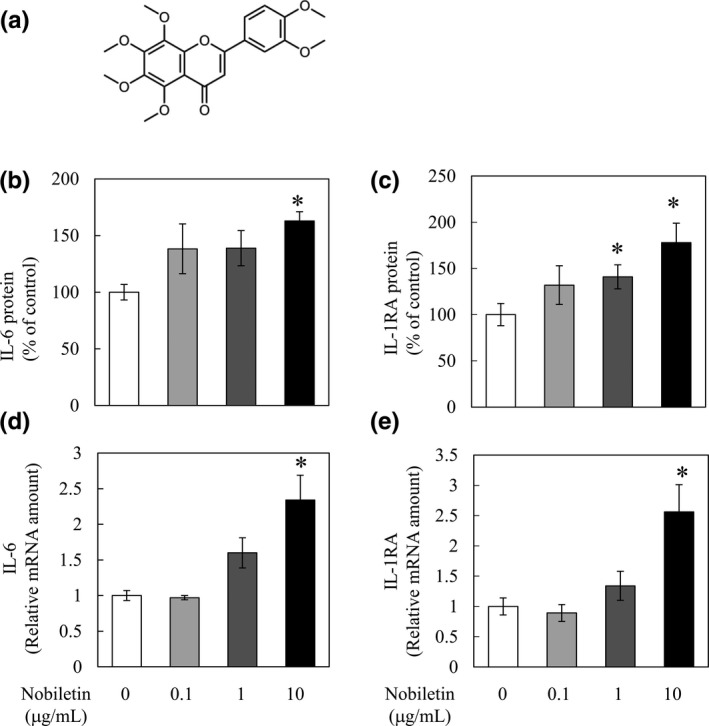
Interleukin‐6 (IL‐6) and IL‐1 receptor antagonist (IL‐1RA) secretion and messenger RNA (mRNA) expression by differentiated C2C12 cells after nobiletin administration. C2C12 cells were incubated for 3 hr in differentiation medium containing 0.1 to 10 μg/ml of nobiletin. After incubation, the culture medium was collected to quantify IL‐6 and IL‐1RA by enzyme‐linked immunosorbent assay (ELISA). Myotubes were collected for IL‐6 and IL‐1RA mRNA expression analysis. The structure of (a) nobiletin. The levels of (b) IL‐6 and (c) IL‐1RA in the culture medium are expressed as the % of the nobiletin‐free group. (d) IL‐6 and (e) IL‐1RA mRNA levels in C2C12 cells. Data are presented as the means ± SEM (*n* = 4). ^*^Significant difference in comparison to the nobiletin‐free group at *p* < .05 by Dunnett's test

Nobiletin (3',4',5,6,7,8‐hexamethoxyflavone) is a well‐known polymethoxyflavone (PMF) (Figure 1a) that is abundantly present in the peels of citrus fruits (Nogata et al., 2006). PMFs have anti‐inflammatory, anti‐obesity, anti‐diabetes, anti‐dementia, and anti‐cancer activities. Recently, we reported that orange peel extract (OPE) containing PMFs suppresses muscle inflammation and damage induced by downhill running (Suzuki et al., 2021a), and that these effects of OPE occur via an increase in IL‐1 receptor antagonist (IL‐1RA) (Suzuki et al., 2021b). In a series of studies, we have found that nobiletin also promotes the secretion of IL‐6 from C2C12 myotubes in addition to IL‐1RA (Suzuki et al., 2021a). However, it remains unclear whether nobiletin increases plasma IL‐6 concentrations during exercise. If so, it may provide an example of a food component that enhances the beneficial effects of exercise via myokines.

In the present study, we investigated the effects of nobiletin on IL‐6 secretion and the mechanism of IL‐6 secretion in C2C12 myotubes. Furthermore, we evaluated whether nobiletin further enhances plasma IL‐6 levels that are increased by exercise in rats.

## 2 MATERIALS AND METHODS

### 2.1 Animals

Female 8‐week‐old Sprague‐Dawley (*SD*) rats were purchased from CLEA Japan, Inc. (Tokyo, Japan); they were housed individually in temperature‐controlled rooms (22°C) and maintained under a constant 12‐hr light–dark cycle at an animal facility. Animals were fed a chow diet and tap water ad libitum. All animal experiments were conducted in 2017, and all protocols for animal procedures were approved by the Animal Care and Use Committee of the University of Tokyo (approval number P17‐065), which are based on the Law for the Humane Treatment and Management of Animals (Law No. 105, October 1, 1973, as amended on June 1, 2020).

### 2.2 Reagents

Nobiletin, epinephrine hydrochloride, and 3‐(isopropylamino)‐1‐[(7‐methyl‐4‐indanyl) oxy]butan‐2‐ol hydrochloride (ICI‐118,551) were purchased from Sigma (St. Louis, MO). 9‐(Tetrahydrofuran‐2‐yl)‐9h‐purin‐6‐amine (SQ22536), N‐[2‐[[3‐(4‐bromophenyl)‐2‐propenyl]amino]ethyl]‐5‐isoquinolinesulfonamide dihydrochloride (H‐89), and anthra(1,9‐cd)pyrazol‐6(2H)‐one (SP600125) were purchased from FUJIFILM Wako Pure Chemical (Osaka, Japan).

### 2.3 Cell cultures and treatment

#### 2.3.1 Cell cultures

The C2C12 mouse myoblast cell line was obtained from the American Type Culture Collection (Manassas, VA). Cells were maintained using 10‐cm dishes (Corning, One Riverfront Plaza, NY) at 37°C under an atmosphere containing 5% CO_2_ in Dulbecco's Modified Eagle's Medium (DMEM) (Thermo Fisher Scientific, Waltham, MA) containing 10% fetal bovine serum (Sigma‐Aldrich), penicillin (100 U/ml; Nacalai Tesque, Kyoto, Japan), and streptomycin (100 μg/ml; Nacalai Tesque). C2C12 myoblasts were plated in 12‐well plates (AGC TECHNO GLASS, Shizuoka, Japan) and confluent myoblasts were differentiated by incubation in differentiation medium containing 2% horse serum (Sigma‐Aldrich) in DMEM for 5 to 7 days.

#### 2.3.2 Cytokine secretion assay using nobiletin or epinephrine alone

Nobiletin dissolved in dimethyl sulfoxide (DMSO) was added to the differentiation medium at a final concentration of 0.1–10 μg/ml. Epinephrine hydrochloride dissolved in distilled water was added to the differentiation medium at a final concentration of 1–100 pg/ml. Differentiated C2C12 cells were incubated at 37°C under an atmosphere containing 5% CO_2_ for 3 hr in differentiation medium containing nobiletin or epinephrine. The final concentration of DMSO was 0.1%. After incubation, the culture medium was collected for the quantification of IL‐6, IL‐1RA, and C‐X‐C motif chemokine ligand 1(CXCL‐1). Cells were collected for IL‐6, IL‐1RA, and CXCL‐1 mRNA (messenger RNA) expression analysis.

#### 2.3.3 Nobiletin and epinephrine combination assay

Differentiated C2C12 cells were incubated for 3 hr in differentiation medium containing 1 μg/ml nobiletin and/or 1 ng/ml epinephrine. The final concentration of DMSO was 0.1%. After incubation, the culture medium was collected for the quantification of IL‐6 and CXCL‐1 by enzyme‐linked immunosorbent assay (ELISA) kits. Cells were collected for IL‐6 and CXCL‐1 mRNA expression analysis and the quantification of cyclic adenosine monophosphate (cAMP) levels.

#### 2.3.4 Inhibition assay of cytokine secretion using inhibitors

Differentiated C2C12 cells were pretreated with the β2‐adrenergic receptor antagonist ICI‐118,551 (1 μg/ml), the adenylyl cyclase (AC) inhibitor SQ22536 (3 μg/ml), the protein kinase A (PKA) inhibitor H‐89 (1 μg/ml), or the c‐jun N‐terminal kinase (JNK) inhibitor SP600125 (3 μg/ml) for 30 min before adding epinephrine (100 ng/ml) or nobiletin (10 μg/ml). The final concentration of DMSO was 0.2%. The culture medium was collected for the quantification of IL‐6 and CXCL‐1 by ELISA kits 3 hr after the addition of epinephrine or nobiletin.

#### 2.3.5 Inhibition assay of cytokine secretion using a small interfering RNA (siRNA) of β2‐adrenergic receptor

Differentiated C2C12 cells were pretreated with the siRNA of β2‐adrenergic receptor or negative control (Thermo Fisher Scientific) for 72 hr and then with epinephrine (100 ng/ml) or nobiletin (10 μg/ml). After 3 hr, the culture medium and cells were collected to quantify IL‐6 or CXCL‐1 in medium and expression levels of β2‐adrenergic receptor in cells.

#### 2.3.6 Evaluation of PKA–cAMP response element binding protein (CREB) pathway activation after nobiletin administration

DifferentiatedC2C12 cells were treated with nobiletin (10 μg/ml). After 3 hr, cells were collected, and quantified expression levels of phosphorylated CREB and total CREB by Western blotting (WB).

### 2.4 Real‐time quantitative polymerase chain reaction (qPCR)

Total RNA was isolated from tissues using ISOGEN (Nippon Gene, Tokyo, Japan) and then purified using a RNeasy Mini Kit (QIAGEN, Hilden, Germany) in accordance with the manufacturers’ instructions. Isolated RNA was reverse‐transcribed to complementary DNA (cDNA) using the High‐Capacity cDNA Reverse Transcription Kit (Thermo Fisher Scientific). Real‐time PCR was performed using the TaqMan Gene Expression Assay (Thermo Fisher Scientific) on a QuantStudio 3 Real‐Time PCR System (Thermo Fisher Scientific). All primers and probes were purchased as TaqMan Gene Expression Assays: IL‐6 (Mm99999064_m1), IL‐1RA (Mm00446186_m1), CXCL‐1 (Mm04207460_m1), and Gapdh (glyceraldehyde 3‐phosphate dehydrogenase) (Mm99999915_g1). Gene expression levels were normalized by Gapdh and expressed as fold change relative to that of the control group.

### 2.5 ELISA

IL‐6 and CXCL‐1 in the culture medium were measured using ELISA kits from R&D systems (Minneapolis, MN), and IL‐1RA was measured using an ELISA kit from Abcam (Cambridge, UK). Plasma epinephrine and norepinephrine were measured using an ELISA kit from ImmuSmol (Bordeaux, France). Plasma IL‐6 was measured using an ELISA kit from Thermo Fisher Scientific, and CXCL‐1 was measured using an ELISA kit from R&D systems.

### 2.6 Western blotting

Cytoplasmic extracts were prepared using Nuclear and Cytoplasmic Extraction Reagents (Thermo Fisher Scientific) according to the manufacturer's instructions. Protein samples were electrophoresed in an Extra PAGE One Precast Gel (5% to 15%; Nacalai Tesque, Kyoto, Japan), transferred to a polyvinylidene difluoride membrane (Merck Millipore, Burlington, MA) at room temperature, and then incubated with Blocking One (Nacalai Tesque) for 1 hr. Antibodies against phospho‐CREB (Ser133), total CREB, and β‐actin were purchased from Cell Signaling Technology (Danvers, MA). After an overnight incubation, the blots were washed three times with a wash buffer (PBS with 0.2% Tween 20) for 15 min each time at room temperature and then incubated for 2 hr with a secondary horseradish peroxidase‐conjugated goat anti‐rabbit antibody (Santa Cruz Biotechnology, Santa Cruz, CA). The blots were then washed three times as described above. Antigen detection was performed with an enhanced chemiluminescence system (GE Healthcare, Chicago, IL). Quantification of the Western blots was performed using FUSION SOLO S (Vilber‐Lourmat, Marne, France).

### 2.7 cAMP assay

The intracellular cAMP level was measured using a cAMP EIA Kit (Cayman Chemical, Ann Arbor, MI).

### 2.8 Treadmill running experiment

Twenty rats were initially acclimated to running on a motor‐driven treadmill at 15 m/min, 0% grade, for 15 min/day for three days during the first week, and then they were used for experiments during the second week. In the second week, the 20 rats were randomly divided into four groups (*n* = 5), i.e., the resting group, treadmill running exercise (Ex) group, nobiletin 5 mg/kg (Nob) group, and Ex +Nob group. Nob and Ex +Nob groups were orally administered nobiletin (5 mg/kg) suspended in a 0.5% carboxymethyl cellulose (CMC)–Na aqueous solution at 10 ml/kg 30 min before treadmill running. The resting and Ex groups were administered only the 0.5% CMC–Na aqueous solution at 10 ml/kg. The Ex and Ex +Nob groups were subjected to running at 20 m/min with a 0% grade on the treadmill for 60 min. The resting and Nob groups were not subjected to running. Animals were anesthetized and euthanized with isoflurane after the treadmill exercise. After euthanasia, abdominal vein blood samples were collected in ethylenediaminetetraacetic acid (EDTA)–2Na tubes for the measurement of plasma IL‐6 and CXCL‐1 levels and EDTA–2Na tubes containing sodium metabisulfite for the measurement of plasma epinephrine and norepinephrine levels, and plasma was stored at −80°C.

### 2.9 Statistical analyses

All statistical analyses were performed using BellCurve for Excel (Version 2.00). In the experiments using C2C12 myotubes with nobiletin and epinephrine administered alone, after one‐way analysis of variance (ANOVA), multiple comparisons with the nobiletin‐free or epinephrine‐free groups were performed using Dunnett's test. In the evaluation of CREB pathway activation experiment, the data were analyzed by unpaired *t*‐test. In the other experiments, the data were analyzed by one‐way ANOVA and a subsequent Tukey–Kramer test to identify differences among groups. In both statistical tests, the significance level was set at *p* < .05.

## 3 RESULTS

### 3.1 Nobiletin increases IL‐6 and IL‐1RA secretion and mRNA expression

We previously reported that nobiletin increases IL‐6 and IL‐1RA secretion from C2C12 myotubes (Suzuki et al., 2021a). To investigate how nobiletin induced the increased secretion of IL‐6 and IL‐1RA from differentiated C2C12 cells, we measured IL‐6 and IL‐1RA mRNA expression in cells in addition to IL‐6 and IL‐1RA concentrations in the medium in the presence of nobiletin at 0.1–10 μg/ml. Nobiletin (10 μg/ml) increased the secretion of IL‐6 from cells and IL‐6 and IL‐1RA mRNA expression (Figure 1b,d,e). Nobiletin (1 and 10 μg/ml) also increased the secretion of IL‐1RA from cells (Figure 1c). These results show that nobiletin increases IL‐6 mRNA levels and induces its secretion from differentiated C2C12 cells, but only at 10 μg/ml.

### 3.2 Epinephrine increases IL‐6 secretion and mRNA expression

**FIGURE 2:**
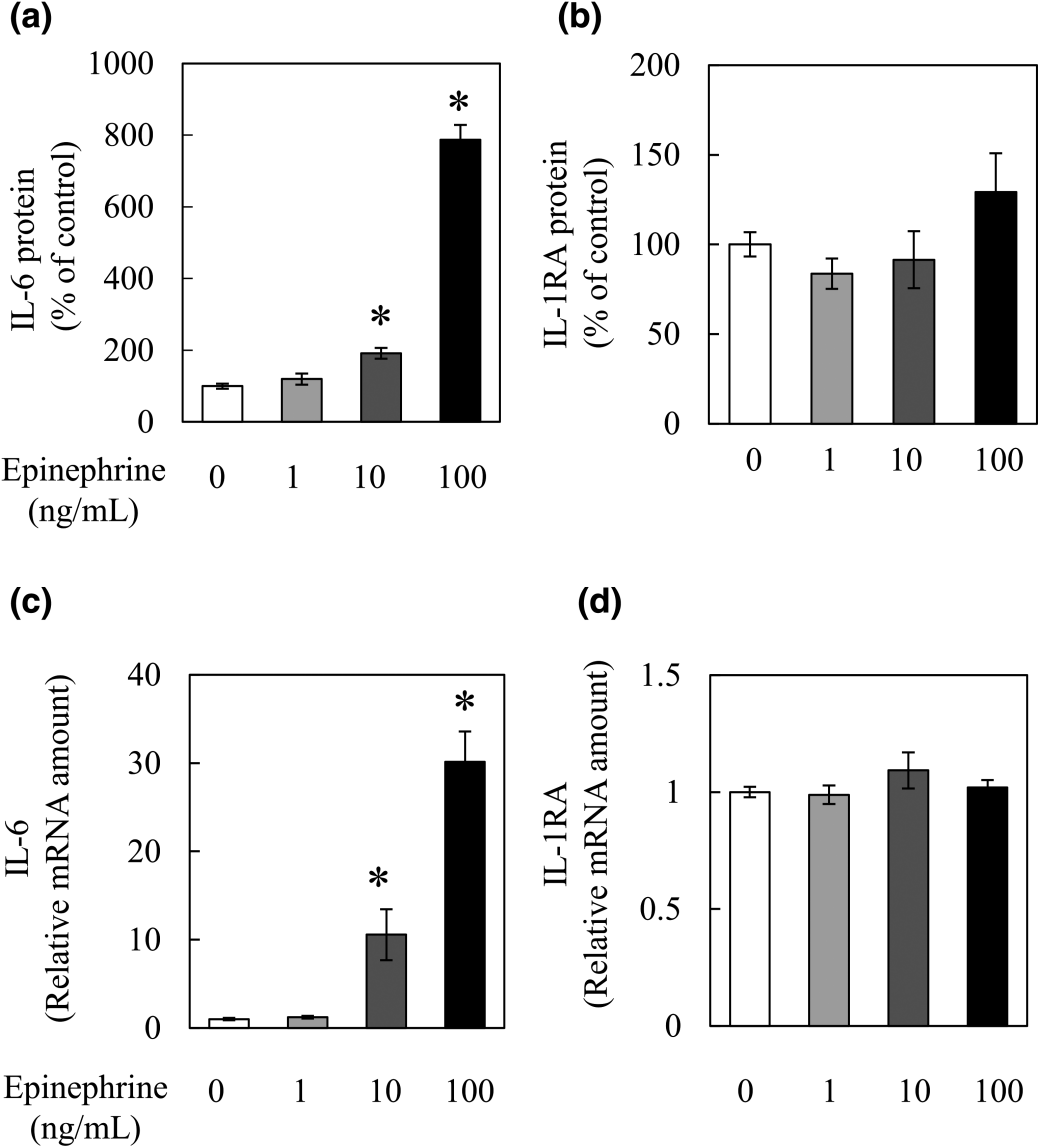
Interleukin‐6 (IL‐6) and IL‐1 receptor antagonist (IL‐1RA) secretion and messenger RNA (mRNA) expression of differentiated C2C12 cells after epinephrine administration. C2C12 cells were incubated for 3 hr in differentiation medium containing 1 to 100 ng/ml epinephrine. After incubation, the culture medium was collected to quantify IL‐6 and IL‐1RA by enzyme‐linked immunosorbent assay (ELISA). Cells were collected for IL‐6 and IL‐1RA mRNA expression analysis. The levels of (a) IL‐6 and (b) IL‐1RA in the culture medium are expressed as the % of the epinephrine‐free group. (c) IL‐6 and (d) IL‐1RA mRNA levels in C2C12 cells. Data are presented as the means ± SEM (*n* = 4). ^*^Significant difference in comparison to the epinephrine‐free group at *p* < .05 by Dunnett's test

Epinephrine is increased by exercise and is reported to increase IL‐6 secretion and mRNA expression (Frost et al., 2004; Koch et al., 1980). To confirm its effects on the secretion of IL‐6 and IL‐1RA as well as the effects of nobiletin, we conducted a similar experiment in the presence of epinephrine at 1–100 ng/ml. Epinephrine (10 and 100 ng/ml) increased the secretion of IL‐6 from cells and IL‐6 mRNA expression (Figure 2a,c). Epinephrine (1–100 ng/ml) did not induce the secretion of IL‐1RA or increase IL‐1RA mRNA (Figure 2b,d). These results show that epinephrine increases IL‐6 mRNA levels and induces its secretion from cells, but only at concentrations of 10 ng/ml and above. Also, IL‐1RA secretion is unrelated to epinephrine in a concentration range of 1–100 ng/ml.

### 3.3 Nobiletin and epinephrine synergistically increase IL‐6 secretion, IL‐6 mRNA, and intracellular cAMP

**FIGURE 3:**
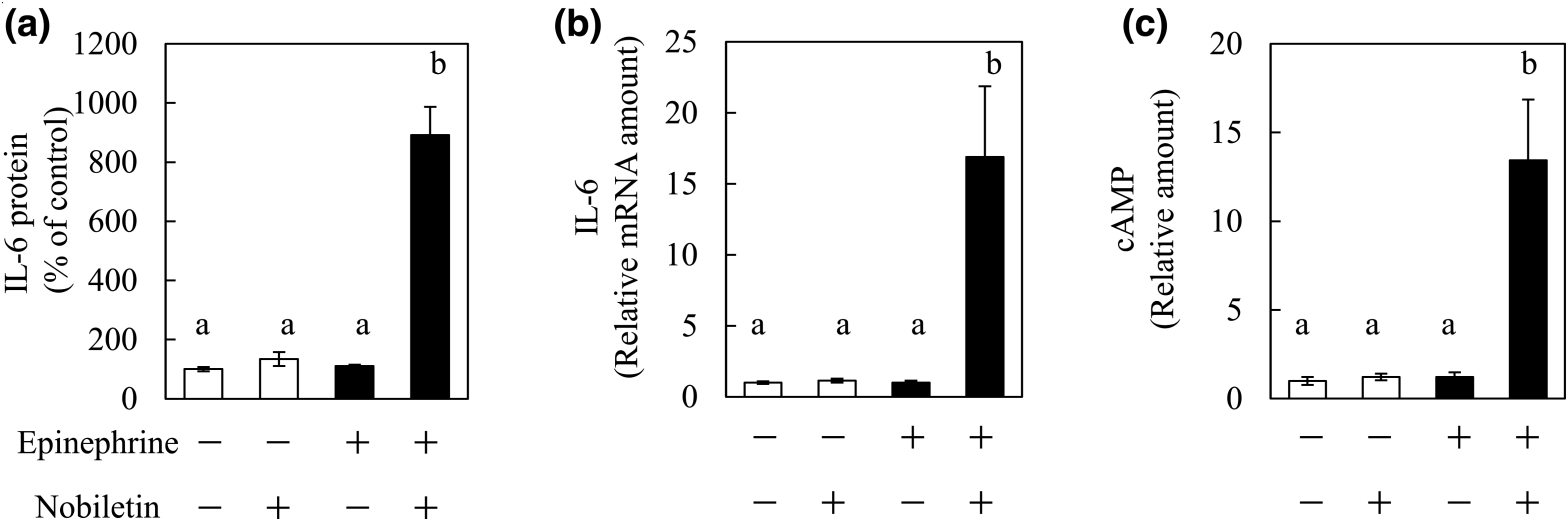
Synergistic effects of nobiletin and epinephrine on Interleukin‐6 (IL‐6) secretion, messenger RNA (mRNA) expression, and cyclic adenosine monophosphate (cAMP) levels. C2C12 cells were incubated for 3 hr in differentiation medium containing 1 μg/ml of nobiletin and/or 1 ng/ml of epinephrine. After incubation, the culture medium was collected to quantify IL‐6 by enzyme‐linked immunosorbent assay (ELISA). Cells were collected for IL‐6 mRNA expression analysis and used to quantify cAMP levels. The levels of (a) IL‐6 in the culture medium are expressed as the % of the control group. (b) IL‐6 mRNA and (c) cAMP levels in C2C12 cells. Data are presented as the means ± SEM (*n* = 4). ^a,b^Values not sharing a common letter differed significantly at *p* < .05 by the Tukey–Kramer test

To determine the effects of a combination of nobiletin and epinephrine, differentiated C2C12 cells were treated with nobiletin (1 μg/ml) and epinephrine (1 ng/ml), which alone do not induce the secretion of IL‐6 or increase IL‐6 mRNA expression at these concentrations. While neither alone induces IL‐6 secretion, IL‐6 secretion was more than eightfold with the combination of nobiletin and epinephrine than without either (Figure 3a). IL‐6 mRNA expression and the intracellular cAMP level showed similar trends (Figure 3b,c). These data suggest that the combination of nobiletin and epinephrine synergistically induces the secretion of IL‐6 from cells along with increased intracellular cAMP levels.

### 3.4 Nobiletin induces IL‐6 secretion via the PKA–CREB pathway

It has been reported that the secretion of IL‐6 from myotubes and cardiomyocytes via β2‐adrenergic receptor stimulation involves activation of the PKA or JNK pathway (Frost et al., 2004; Szabo‐Fresnais et al., 2010). To clarify the mechanism behind the synergy of nobiletin and epinephrine, we investigated their effects on IL‐6 secretion using an antagonist or inhibitors related to β2‐adrenergic receptor signaling.

**FIGURE 4:**
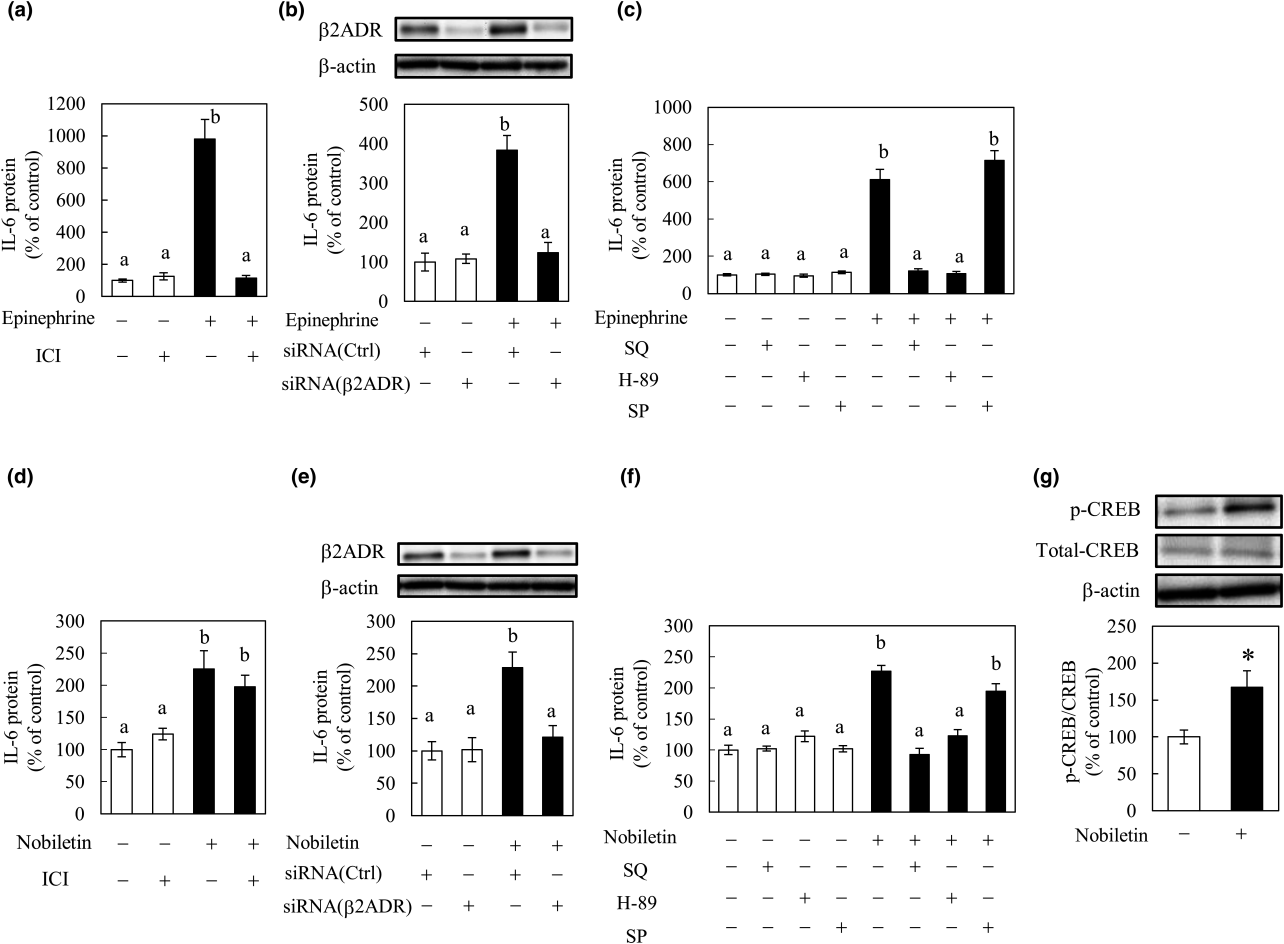
Effects of various inhibitors or small interfering RNA (siRNA) on Interleukin‐6 (IL‐6) secretion from differentiated C2C12 cells by nobiletin and epinephrine. C2C12 cells were pretreated with the β2‐adrenergic receptor antagonist ICI‐118,551 (1 μg/ml) for 30 min and then with (a) epinephrine (100 ng/ml) or (d) nobiletin (10 μg/ml). After 3 hr, the culture medium was collected to quantify IL‐6 by enzyme‐linked immunosorbent assay (ELISA). C2C12 cells were pretreated with the siRNA of β2‐adrenergic receptor for 72 hr and then with (b) epinephrine (100 ng/ml) or (e) nobiletin (10 μg/ml). After 3 hr, the culture medium and cells were collected to quantify IL‐6 in medium by ELISA and expression levels of β2‐adrenergic receptor in cells by Western blotting (WB). C2C12 cells were pretreated with the adenylyl cyclase (AC) inhibitor SQ22536 (3 μg/ml), the protein kinase A (PKA) inhibitor H‐89 (1 μg/ml), or the c‐jun N‐terminal kinase (JNK) inhibitor SP‐600125 (3 μg/ml) for 30 min before adding (c) epinephrine (100 ng/ml) or (f) nobiletin (10 μg/ml). After 3 hr, the culture medium was collected to quantify IL‐6 by ELISA. The levels of IL‐6 in the culture medium are expressed as the % of the control group. (g) C2C12 cells were treated with nobiletin (10 μg/ml). After 3 hr, cells were collected, and quantified expression levels of phosphorylated cAMP response element binding protein (CREB) and total CREB by Western blotting (WB). Data are presented as the means ± SEM (*n* = 6). ^a,b^Values not sharing a common letter differed significantly at *p* < .05 by the Tukey–Kramer test.^*^Significant difference in two groups at *p* < .05 by unpaired *t*‐test

The β2‐adrenergic receptor antagonist ICI‐118,551 and siRNA of β2‐adrenergic receptor suppressed the IL‐6 secretion induced by epinephrine (Figure 4a,b). Both the AC inhibitor SQ22536 and the PKA inhibitor H‐89 also reduced IL‐6 secretion induced by epinephrine (Figure 4c), which confirms that epinephrine acts on β2‐adrenergic receptor to elevate IL‐6 secretion via AC and PKA. ICI‐118,551 did not suppress IL‐6 secretion induced by nobiletin (Figure 4d), but siRNA suppressed this secretion (Figure 4e). SQ22536 and H‐89 decreased IL‐6 secretion promoted by nobiletin (Figure 4f). Nobiletin promoted phosphorylation of CREB (Figure 4g). These results suggest that nobiletin is not a ligand for β2‐adrenergic receptor, but nobiletin would promote IL‐6 secretion by activating the PKA–CREB pathway through allosteric binding for β2‐adrenergic receptor. The JNK inhibitor SP‐600125 did not affect IL‐6 secretion promoted by either epinephrine or nobiletin (Figure 4c,f). A relationship between the JNK pathway and IL‐6 secretion was not observed under the conditions of this study.

### 3.5 Nobiletin enhances the exercise‐induced increase in plasma IL‐6 levels in rats

**FIGURE 5:**
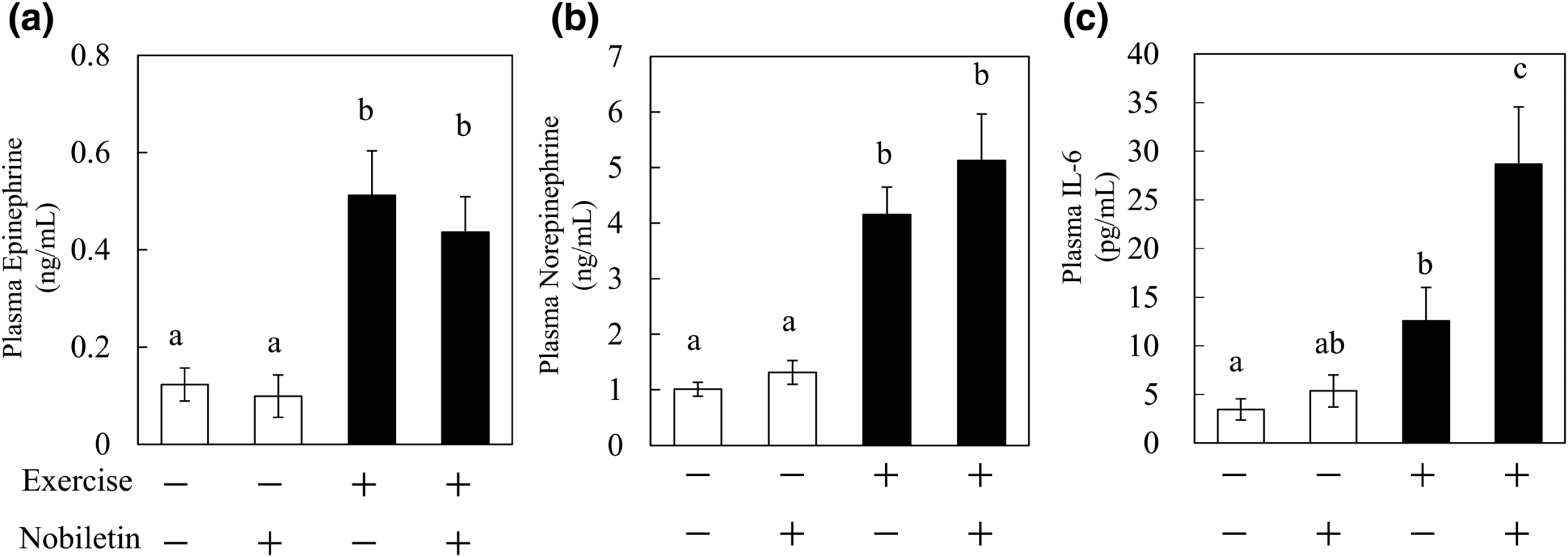
Synergistic increase in plasma Interleukin‐6 (IL‐6) levels by treadmill running and nobiletin administration. Twenty rats were randomly divided into four groups (*n* = 5): resting group, treadmill running exercise (Ex) group, nobiletin 5 mg/kg (Nob) group, and Ex +Nob group. Nob and Ex +Nob groups were orally administered nobiletin 30 min before treadmill running. The Ex and Ex +Nob groups were subjected to running at 20 m/min with a 0% grade on a treadmill for 60 min. The resting and Nob groups were not subjected to running. Plasma levels of (a) epinephrine, (b) norepinephrine, and (c) IL‐6 were measured after treadmill running. Data are presented as the means ± SEM of five rats. ^a,b,c^Values not sharing a common letter differed significantly at *p* < .05 by the Tukey–Kramer test

Treadmill running increases plasma epinephrine and norepinephrine levels (Koch et al., 1980). Therefore, we expected that nobiletin and the exercise‐induced elevation of epinephrine and norepinephrine would synergistically increase IL‐6 secretion from skeletal muscle in rats. We confirmed that plasma epinephrine and norepinephrine levels were increased by treadmill running, to more than 0.4 and 4 ng/ml, respectively (Figure 5a,b). The administration of nobiletin (5 mg/kg) did not affect either plasma epinephrine or norepinephrine with or without treadmill running (Figure 5a,b). While plasma IL‐6 levels were increased by treadmill running (Figure 5c) as expected, the administration of nobiletin did not change plasma IL‐6 levels without exercise. Notably however, the plasma IL‐6 level was approximately two times higher with nobiletin than without it following treadmill running, suggesting that the combination of running and nobiletin had a synergistic effect on the increase in plasma IL‐6 in rats.

### 3.6 CXCL‐1 secretions from differentiated C2C12 cells are induced synergistically by nobiletin and epinephrine, and the exercise‐induced increase in plasma levels is enhanced by nobiletin administration

**FIGURE 6:**
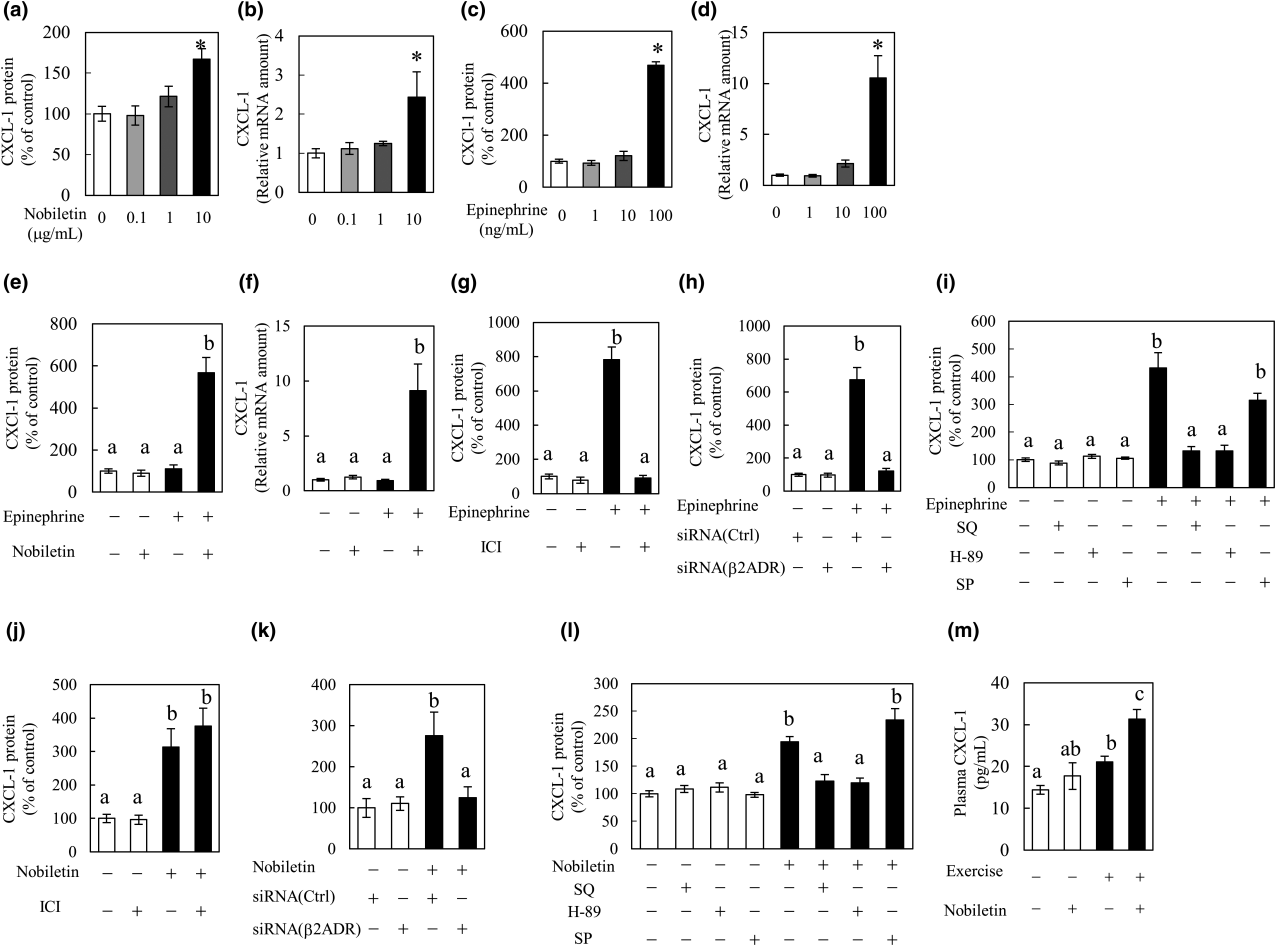
Synergistic effect of nobiletin and epinephrine on C‐X‐C motif chemokine ligand 1 (CXCL‐1) secretion in vitro and nobiletin and exercise on plasma CXCL‐1 increase in vivo. (a–d) C2C12 cells were incubated for 3 hr in differentiation medium containing 0.1 to 10 μg/ml of nobiletin or 1 to 100 ng/ml of epinephrine. After incubation, the culture medium was collected to quantify (a, c) CXCL‐1 by enzyme‐linked immunosorbent assay (ELISA). C2C12 cells were collected for (b, d) CXCL‐1 mRNA (messenger RNA) expression analysis. Data are presented as the means ± SEM (*n* = 4). ^*^ Significant difference in comparison to the nobiletin‐free or epinephrine‐free group at *p* < .05 by Dunnett's test. (E‐F) C2C12 cells were incubated for 3 hr in differentiation medium containing 1 μg/ml of nobiletin and/or 1 ng/ml of epinephrine. After incubation, the culture medium was collected to quantify (E) CXCL‐1 by ELISA. Cells were collected for (f) CXCL‐1 mRNA expression analysis. The levels of CXCL‐1 in the culture medium are expressed as the % of the control group. Data are presented as the means ±SEM(*n* = 6). ^a,b^Values not sharing a common letter differed significantly at *p* < .05 by the Tukey–Kramer test. (g, j) C2C12 cells were pretreated with the β2‐adrenergic receptor antagonist ICI‐118,551 (1 μg/ml) for 30 min and then with (g) epinephrine (100 ng/ml) or (i) nobiletin (10 μg/ml). After 3 hr, the culture medium was collected to quantify CXCL‐1 by ELISA. The levels of CXCL‐1 in the culture medium are expressed as the % of the control group. Data are presented as the means ± SEM (*n* = 4). ^a,b^Values not sharing a common letter differed significantly at *p* < .05 by the Tukey–Kramer test. (h, k) C2C12 cells were pretreated with the siRNA of β2‐adrenergic receptor for 72 hr and then with (b) epinephrine (100 ng/ml) or (e) nobiletin (10 μg/ml). After 3 hr, the culture medium and cells were collected to quantify CXCL‐1 in medium by ELISA. Data are presented as the means ± SEM (*n* = 6). ^a,b^Values not sharing a common letter differed significantly at *p* < .05 by the Tukey–Kramer test. (i, l) C2C12 cells were pretreated with the adenylyl cyclase (AC) inhibitor SQ22536 (3 μg/ml), the protein kinase A (PKA) inhibitor H‐89 (1 μg/ml), or the c‐jun N‐terminal kinase (JNK) inhibitor SP‐600125 (3 μg/ml) for 30 min before adding (h) epinephrine (100 ng/ml) or (j) nobiletin (10 μg/ml). After 3 hr, the culture medium was collected to quantify CXCL‐1 by ELISA. The levels of CXCL‐1 in the culture medium are expressed as the % of the control group. Data are presented as the means ± SEM (*n* = 6). ^a,b^Values not sharing a common letter differed significantly at *p* < .05 by the Tukey–Kramer test. (k) Twenty rats were randomly divided into four groups (*n* = 5), i.e., the resting group, treadmill running exercise (Ex) group, nobiletin 5 mg/kg (Nob) group, and Ex +Nob group. Nob and Ex +Nob groups were orally administered nobiletin 30 min before treadmill running. The Ex and Ex +Nob groups were subjected to running at 20 m/min with a 0% grade on the treadmill for 60 min. The resting and Nob groups were not subjected to running. Plasma levels of (l) CXCL‐1 were measured after treadmill running. Data are presented as the means ± SEM (*n* = 5).^a,b,c^Values not sharing a common letter differed significantly at *p* < .05 by the Tukey–Kramer test

CXCL‐1 is a myokine that is secreted from skeletal muscle by epinephrine stimulation, similar to IL‐6 (Mattingly et al., 2017). We expected that the regulation of CXCL‐1 secretion is similar to that of IL‐6 secretion, and we conducted similar experiments as described above on CXCL‐1. The tendency of changes of CXCL‐1 secretion was broadly similar to that of IL‐6 in vitro and in vivo. Nobiletin (10 μg/ml) and epinephrine (100 ng/ml) each increased CXCL‐1 secretion and mRNA expression (Figure 6a‐d), and nobiletin and epinephrine together synergistically increased CXCL‐1 secretion and CXCL‐1 mRNA expression (Figure 6e,f). ICI‐118,551 and siRNA of β2‐adrenergic receptor suppressed the CXCL‐1 secretion induced by epinephrine (Figure 6g,h). ICI‐118,551 did not suppress the CXCL‐1 secretion by nobiletin, but siRNA suppressed (Figure 6j,k). SQ22536 and H‐89 reduced CXCL‐1 secretion promoted by epinephrine and nobiletin (Figure 6i,l). Plasma CXCL‐1 levels were increased by treadmill running, and this was enhanced by oral administration of 5 mg/kg nobiletin (Figure 6m). These results indicate that CXCL‐1 secretion is synergistically increased by nobiletin and epinephrine and that the mechanism of CXCL‐1 secretion by nobiletin involves the PKA–CREB pathway as well as IL‐6.

## 4 DISCUSSION

Nobiletin has anti‐inflammatory, anti‐obesity, anti‐diabetes, anti‐dementia, and anti‐cancer activities (Huang et al., 2016). Previously, we found that it has the potential to induce the secretion of IL‐6 and IL‐1RA from C2C12 myotubes, but the details remain unclear. In the present study, we found that nobiletin increases the secretion and the mRNA expression of both IL‐6 and IL‐1RA in C2C12 myotubes (Figure 1a‐d).

It is well known that exercise elevates plasma epinephrine (Fischer, 2006), which increases IL‐6 secretion from myotubes and skeletal muscle (Frost et al., 2004; Mattingly et al., 2017). We confirmed that epinephrine promotes IL‐6 secretion and IL‐6 mRNA expression in C2C12 myotubes in this study (Figure 2a,c). However, IL‐1RA secretion and mRNA expression levels were not affected by its administration, unlike IL‐6 (Figure 2b,d), suggesting that IL‐1RA secretion is unrelated to the pathway affected by epinephrine in a range of 1–100 ng/ml.

Since both nobiletin and epinephrine alone have an effect of inducing IL‐6 secretion, we next examined the combined effect of these two compounds. We used nobiletin at 1 μg/ml and epinephrine at 1 ng/ml, a concentration at which neither of which increased the IL‐6 secretion or IL‐6 mRNA expression alone. IL‐6 secretion, IL‐6 mRNA expression, and intracellular cAMP levels were clearly elevated with the combination of nobiletin and epinephrine (Figure 3a–c). The synergistic effects of nobiletin as a food ingredient and epinephrine as a hormone are interesting, and we attempted to clarify the mechanism of this synergy.

We investigated the mechanism using an antagonist or inhibitors or siRNA related to β2‐adrenergic receptor signaling. Because increases in IL‐6 secretion and mRNA expression via β2‐adrenergic receptor involve the cAMP–PKA signaling pathway (Kolmus et al., 2014; Szabo‐Fresnais et al., 2010), the present data expectedly show that these increases by epinephrine were suppressed by an β2‐adrenergic receptor inhibitor, a siRNA, an AC inhibitor, and a PKA inhibitor (Figure 4a,b,c). However, the β2‐adrenergic receptor inhibitor did not suppress IL‐6 secretion by nobiletin (Figure 4d), but β2‐adrenergic receptor knockdown by siRNA suppressed (Figure 4e). These results suggest that nobiletin is not a ligand for β2‐adrenergic receptor but an activator via allosteric binding, suggesting that epinephrine and nobiletin activate β2‐adrenergic receptor from different sites, resulting in synergistic IL‐6 secretion.

Nobiletin has a protective effect on hippocampal neurons of SD rats and promotes neuronal differentiation in PC12 neuronal cells through activation of the PKACREB pathway (Matsuzaki et al., 2006; Nagase et al., 2005). Our results that IL‐6 secretion by nobiletin was inhibited by AC and PKA inhibitors (Figure 4f) and that nobiletin promoted CREB phosphorylation (Figure 4g) were consistent with these previous reports.

A relationship between the JNK pathway and IL‐6 secretion was not observed under the conditions in this study (Figure 4b,d). This is consistent with a report that the JNK pathway is not activated by isoproterenol, an agonist of β2‐adrenergic receptor using C2C12 myotubes (Kolmus et al., 2014). However, there is a contrasting report indicating that JNK inhibition suppresses IL‐6 secretion by epinephrine (Frost et al., 2004). The details are therefore still unclear, so further studies concerning the involvement of the JNK pathway are needed.

Next, we investigated the in vivo effects of a combination of nobiletin and epinephrine on IL‐6 secretion. Plasma epinephrine and norepinephrine increase during exercise (Koch et al., 1980). We previously reported that oral administration of OPE containing nobiletin (5 mg/kg) does not increase plasma IL‐6 levels under nonexercise conditions (Suzuki et al., 2021a). In the present study, we expected the elevation of plasma epinephrine levels by exercise and investigated whether nobiletin could enhance plasma IL‐6 levels under exercise conditions. We found that treadmill running elevated plasma epinephrine and norepinephrine levels (Figure 5a,b), but nobiletin did not affect their levels with or without treadmill running (Figure 5a,b). These results show that nobiletin at 5 mg/kg did not have the ability to increase plasma epinephrine or norepinephrine. Under the same conditions, nobiletin did not increase plasma IL‐6 levels without running, but with running it increased plasma IL‐6 levels by approximately twofold compared to without running (Figure 5c). These results indicate that the combination of running and nobiletin has a synergistic effect on increasing plasma IL‐6 in rats. It remains unclear whether this in vivo phenomenon is explained by the in vitro synergistic effect of nobiletin and epinephrine in C2C12 myotubes as described above, but we consider it plausible in terms of their concentrations. We used nobiletin at 1 μg/ml and epinephrine at 1 ng/ml in the in vitro experiment (Figure 3). Plasma concentrations of nobiletin on the order of microgram/milliliter (μg/ml) are thought to be achievable, because the maximum plasma concentration (Cmax) of nobiletin in rats reaches 56 ng/ml when nobiletin at only 0.17 mg/kg body weight is given (Kotani et al., 2015). We quantified epinephrine and norepinephrine in this study, and their plasma concentrations (epinephrine, >0.4 ng/ml; norepinephrine,> 4 ng/ml) are comparable to those in the in vitro experiment. Therefore, it is possible that a situation similar to that of the in vitro experiment occurred in the skeletal muscle of rats in this study.

Plasma epinephrine and norepinephrine levels increased by treadmill running, but nobiletin had no effect on their plasma levels (Figure 5b,c). The catecholamines increased by treadmill running and nobiletin may act synergistically on skeletal muscle, leading to an increase in plasma IL‐6 levels. These results suggest that nobiletin may enhance the beneficial effects of exercise via IL‐6. However, IL‐6 is also secreted from organs other than skeletal muscle. Therefore, further studies are needed on the specific contribution of skeletal muscle to the increase in plasma IL‐6 levels by nobiletin.

CXCL‐1 is a small peptide belonging to the CXC chemokine family (Pedersen et al., 2012). In skeletal muscle, CXCL‐1 promotes lipid oxidation and promotes myogenesis from muscle satellite cells (Masuda et al., 2018; Pedersen et al., 2012). Similar to IL‐6, CXCL‐1 is secreted from skeletal muscles by epinephrine (Mattingly et al., 2017). Our results, which are consistent with those of previous studies, showed the promotion of CXCL‐1 secretion and increase in CXCL‐1 mRNA expression by epinephrine (Figure 6c,d). Nobiletin also showed similar effects (Figure 6a,b). Similar to IL‐6, CXCL‐1 secretion from myotubes was promoted and CXCL‐1 mRNA was synergistically increased by the combination of epinephrine and nobiletin (Figure 6e,f). Furthermore, we investigated the mechanism of increased CXCL‐1 secretion by epinephrine and nobiletin, and clarified that they are regulated by the PKA–CREB pathway, similar to IL‐6 (Figure 6g–l). The increase in plasma CXCL‐1 levels by treadmill running was also enhanced by nobiletin (Figure 6m). These results suggest that nobiletin may enhance the beneficial effects of exercise such as the anti‐obesity effect and skeletal muscle hypertrophy, by further increasing plasma IL‐6 and CXCL‐1 levels that increase with exercise.

In the present study, we showed that nobiletin further enhances plasma IL‐6 and CXCL‐1 levels that are increased by treadmill running. This may be because of synergistic activation of the PKA pathway by increased plasma catecholamines after exercise and by nobiletin administration, which promotes IL‐6 and CXCL‐1 secretion from skeletal muscle into blood.

## CONFLICT OF INTEREST

No potential conflict of interest was reported by the authors.

## AUTHOR CONTRIBUTION

**Toshihide Suzuki:** Conceptualization (lead); Data curation (lead); Formal analysis (lead); Investigation (lead); Methodology (lead); Project administration (equal); Software (equal); Validation (equal); Visualization (equal); Writing – original draft (lead); Writing – review & editing (equal). **Makoto Shimizu:** Conceptualization (equal); Data curation (equal); Formal analysis (equal); Funding acquisition (lead); Methodology (equal); Project administration (equal); Resources (equal); Supervision (equal); Validation (equal); Visualization (equal); Writing – review & editing (lead). **Yoshio Yamauchi:** Data curation (equal); Project administration (equal); Supervision (equal); Writing – review & editing (equal). **Ryuichiro Sato:** Conceptualization (equal); Project administration (lead); Supervision (equal); Writing – review & editing (equal).

## ETHICAL APPROVAL

All animal experiments were conducted in 2017, and all protocols for animal procedures were approved by the Animal Care and Use Committee of the University of Tokyo (approval number P17‐065), which are based on the Law for the Humane Treatment and Management of Animals (Law No. 105, October 1, 1973, as amended on June 1, 2020).
